# Learning and Transfer: A Perspective From Action Video Game Play

**DOI:** 10.1177/09637214241287171

**Published:** 2024-12-05

**Authors:** Daphne Bavelier, C. Shawn Green

**Affiliations:** 1 Faculty of Psychology and Education Science, University of Geneva; 2 Fondation Campus Biotech, Geneva, Switzerland; 3 Department of Psychology, University of Wisconsin–Madison

**Keywords:** learning, transfer, video game, attention

## Abstract

A growing body of research documents the positive impact that action video game play has on a range of cognitive skills. Such a result, in which training on one task promotes a broad variety of benefits, is a rarity in the cognitive training domain. Instead, the more typical result is that training on one task promotes benefits on that task alone with only limited transfer to untrained tasks. We have proposed that action video game play promotes broad generalization by first enhancing attentional control abilities. This in turn allows for more information to be accrued as one experiences a new task and thus faster learning of that new task. Possible theoretical and practical considerations of such a view are discussed.

The ease with which humans learn and adapt has long been recognized. Given sufficient training time, appropriately titrated levels of difficulty, and suitable spacing between training opportunities, humans tend to show improvements on most tasks. A vexing issue in the field of training, however, is that these behavioral enhancements frequently fail to extend beyond the bounds of the trained task. Examples of extreme learning specificity can be found in virtually every subdomain within psychology, spanning educational psychology, social psychology, developmental psychology, clinical psychology, and human factors just to name a few. For instance, in the perceptual domain, participants can be trained to successfully make ever finer judgments about how well two vertical lines are aligned through repeated practice. However, if those lines are then rotated to be horizontal instead, participants return all the way to untrained levels of performance when asked to judge horizontal, instead of vertical, alignment (Fahle & Poggio, 2002). Similarly, albeit in a study in the totally different domain of human memory, Chase and Simon (1973) showed that experienced chess players remembered up to four times more pieces than beginners chess players if the pieces were set up in a real-game configuration. However, if the pieces were arranged in a totally random configuration, the experienced and beginner chess players were equivalent in terms of their recall. Such specificity of learning represents a significant obstacle to using behavioral training for real-world good because for all practical considerations—from education to patient rehabilitation—generalization beyond the exact task used for training is necessary to ensure daily life impact. A key question then concerns the possibility of identifying or designing training regimens that enhance performance more broadly.

Although not at all designed as, or meant to be, “cognitive training regimens,” the potentially positive impact that playing entertainment-based video games could have on cognitive function was recognized from quite early on (Greenfield et al., 1994). This domain of research significantly accelerated with the coming of games that provided a consistent and heavy load on perceptual, cognitive, and motor function, in particular first- and third-person shooter games, which we refer to here as “action video games” (AVGs; for more discussion of other game types that partially share both mechanics and behavioral outcomes with first- and third-person shooter games, see Dale et al., 2020). Research linking AVG play and cognitive enhancement has utilized two main and complementary methodological approaches. Studies using cross-sectional methods, contrasting performance of self-declared habitual AVG players with that of individuals with little to no video game experience, have shown enhanced performance in AVG players on a variety of measures of cognition. Critically, none of these measures look anything like AVGs. Instead, they are standard psychophysical tasks using simple lines, shapes, letters, and so on. For example, AVG players have been found to have better vision, as indexed by better contrast sensitivity, superior crowding acuity, and less temporal masking compared with non–video game players (Berard et al., 2015; Li et al., 2009). AVG players also exhibit enhanced attentional control, being more efficient at searching for an object within a cluttered scene or redirecting attention to where it is needed for the task when distracted (Chisholm & Kingstone, 2012; Hubert-Wallander et al., 2011) and enhanced multitasking abilities, being able to perform two different tasks together or in rapid succession (Strobach et al., 2012). Finally, benefits have also been noted in a range of spatial tasks such as those that require mentally rotating objects in one’s head (Spence & Feng, 2010). Such cross-sectional studies are valuable in a number of distinct ways—from providing socially relevant information about the cognition of individuals who choose to play AVGs to potentially identifying useful selection criteria for high-level gamers (e.g., esports athletes) and providing pointers as to which cognitive domains the act of playing AVGs may be impacting. However, per the well-known adage, such correlational designs cannot themselves be used to infer a causal link between playing AVGs and enhanced cognition. Indeed, from the correlational studies alone, it would not be possible to tell whether AVGs cause enhancements in cognition or if instead individuals who are born with better cognition or who are more motivated to achieve in lab tasks naturally seek out and play AVGs.

To infer a causal link between play and cognitive enhancement, intervention studies are needed (i.e., randomized controlled trials; see Green et al., 2019; Fig. 1). In such designs, participants’ performance is evaluated before and after being randomly assigned and trained on one of two types of video games, either an AVG (experimental group) or a non-AVG (control group). For the experimental game, first-person shooter games have typically been used. For the control game, researchers have usually selected commercially successful games that use game play mechanics that differ from those of AVGs (in particular that lack speeded decision-making, cluttered visual scenes, and so on; examples include life-simulation games, such as those from *The Sims* series, or business-simulation games, such as those from the *Tycoon* series).

**Fig. 1.**
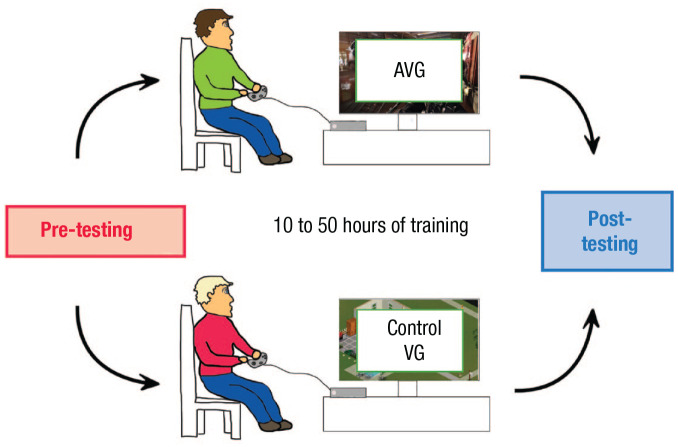
Design of intervention studies contrasting the impact of AVGs versus control video games on cognition. Individuals who play minimal video games are recruited and first pretested on the cognitive measure(s) of interest. They are then assigned to play either an AVG or a control video game (always another successful entertainment video game). The duration of training in the literature ranges from around 10 hr to up to 50 hr, with individual sessions usually lasting around 1 hr. Some studies have had individuals do their training in the lab, whereas others have had participants train at home. Finally, at least 24 hr after the final gaming session, participants return to the lab and complete the same cognitive measure(s) as at pretest. The critical measure is thus whether those in the AVG group show larger improvements from pretest to posttest than those in the control group. AVG = action video game.

Across numerous intervention studies, the AVG-trained group has been reported to improve more than the control-trained group from pre- to posttest for a similar set of cognitive functions, as described above for cross-sectional studies. Meta-analytic work indicates an overall reliable effect (Hedges’s *g*) in the range of 0.3 to 0.35 (Bediou et al., 2018, 2023; however, as is true for all behavioral interventions, not all studies have seen a benefit; see, e.g., Boot et al., 2011). Importantly, in addition to demonstrating a causal role of AVG play in augmenting cognitive function, this research design also strongly highlights the fact that not all video games have the same impact on cognition as AVGs (i.e., because another entertainment video game is explicitly used as a “control,” thus calling for caution when talking about the impact of video games in general on behavior).

## Attentional Control as a Mechanism of Action for Broad Cognitive Enhancements

Given the scope and scale of tasks on which AVGs/individuals trained on AVGs have shown enhancement, one major line of research has focused on addressing whether these broad outcomes can be understood under a common framework. Here, researchers have built on a rich prior literature suggesting that attentional control, which is the ability to flexibly allocate processing resources as task demands change while at the same time staying focused on the task at hand and ignoring sources of noise or distraction, is key to performance enhancement. For example, the proposal that attentional control facilitates performance in a variety of domains is central to many models of executive functions (Diamond, 2013), whereby attentional control, inhibitory processes, and cognitive flexibility are seen as key enablers of performance on most cognitive tasks. Accordingly, a large body of work from education science to perceptual learning has documented the role of executive functions in enhanced performance. For instance, Ahissar and Hochstein (1997) proposed attention to be central for abstracting across task requirements and developing higher levels of representation, thus counteracting the high specificity so frequently seen to arise via learning. By downplaying irrelevant information and highlighting task-relevant information, attentional control also acts as a filter on the information that guides task-related behavior. For these reasons, attentional control has received the most support as a mechanism of action for the relatively broad changes in cognition induced by AVG play (Bavelier & Green, 2019), consistent with this prior work. Indeed, many studies on AVGs have documented enhanced attentional control over space, across time, and to objects.

The key role of attentional control in explaining the impact of AVG play has also been seen in the (much more limited) neurophysiology literature. For instance, studies using brain imaging techniques such as visually evoked potentials have indicated that AVG players more efficiently suppress irrelevant, potentially distracting information and do so despite testing in controlled, non-video-game-like situations (Krishnan et al., 2013; Mishra et al., 2011). Similarly, other work has examined the extent to which better performance arising from AVG experience is more strongly related to changes in early, sensory areas or in later frontal and parietal brain areas. Although there has been limited evidence for changes in early sensory cortices, a few functional MRI studies have reported group differences between AVG players compared with non–video game players in recruitment and connectivity within frontal and parietal areas and their cross-talk with earlier visual areas (Föcker et al., 2018). Together this is most consistent with shifts in brain areas focused on prioritizing what information to process through a combination of both task-relevant information enhancements and distractor suppression (Föcker et al., 2023).

## From Enhancements in Attentional Control to Learning to Learn

**Fig. 2.**
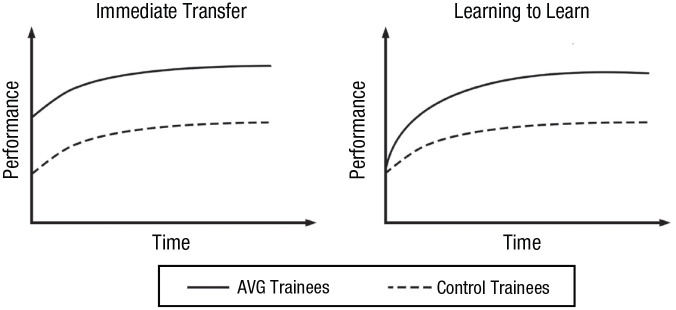
Immediate transfer versus “learning to learn.” Under “immediate transfer,” AVG trainees would outperform control trainees at posttest by a constant amount and do so from the very first trial of a new task. Under “learning to learn,” the difference between AVG and control trainees grows as the task is repeated and learning proceeds. Note that these possibilities are not necessarily mutually exclusive (i.e., it is possible for training to produce both immediately realized benefits on a new task and a boost to learning of the task). AVG = action video game.

One knock-on impact of enhanced attentional control is that it should serve to facilitate the learning of task-relevant information. Accordingly, Zhang et al. (2021) proposed that if a form of training enhances attentional control, the benefits of that training should manifest predominantly as “learning to learn” rather than immediate transfer. This is because the successful deployment of attention requires some minimal understanding of the task demands, which largely cannot be known on the very first trial of a new task. In short, even if someone has enhanced attention control, without knowing when and to what targets to deploy attention to that enhanced attention control cannot result in enhanced task performance from the get-go. Instead, what enhanced attention control will allow for is quicker learning of those task demands and increasingly adaptive deployment of attention based on those task demands (Bavelier et al., 2012). Thus, AVG-related enhancements should manifest as faster learning of new tasks (learning to learn) rather than immediate enhancements (immediate transfer; Fig. 2).

**Fig. 3.**
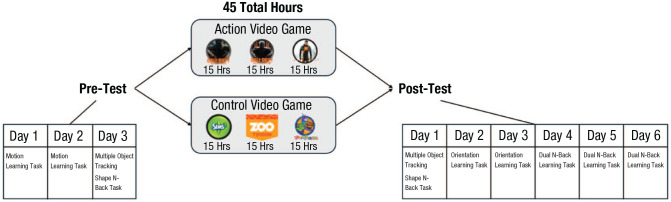
Summary of the design of the true experiment to assess “learning to learn.” To determine whether a form of training has produced “learning to learn,” it is critical that participants be evaluated on new learning tasks after their training. This design begins similarly to that seen in Figure 1, with the exception that one of the tasks is meant to assess pretest/baseline perceptual learning ability (and so takes place over multiple days). Participants then complete training as in Figure 1 before being assessed on new learning tasks at posttest (e.g., here an orientation learning task and a dual *n*-back working memory learning task). The critical questions are thus with respect to whether the action video game trainees and control trainees show immediate differences on the tasks at posttest (which would indicate immediate transfer) and/or if they show differences in the rate of learning or estimated asymptotic level of performance (which would indicate learning to learn).

Critically, evaluating learning to learn as a possible outcome required a shift in methodological approach away from task measures that average over a small numbers of trials toward tasks in which learning can be more easily evaluated (Fig. 3). Indeed, when a single outcome measure (like an average) is extracted from a short task, it would be easy to either miss the learning-to-learn mechanism or else misinterpret it as immediate transfer (Kattner et al., 2017). For example, Li et al. (2009) established that AVG training resulted in enhanced vision utilizing a reasonably short visual perception task from which a single measure of overall performance was extracted. The authors interpreted this result as being indicative of immediate transfer, consistent with nearly all work in the field at the time. However, Zhang et al. (2021) reinterpreted this result as reflecting learning to learn rather than immediate transfer. Critically, such a reinterpretation was conditioned on multisession learning-based measures being used at posttest assessments rather than a single assessment.

**Fig. 4.**
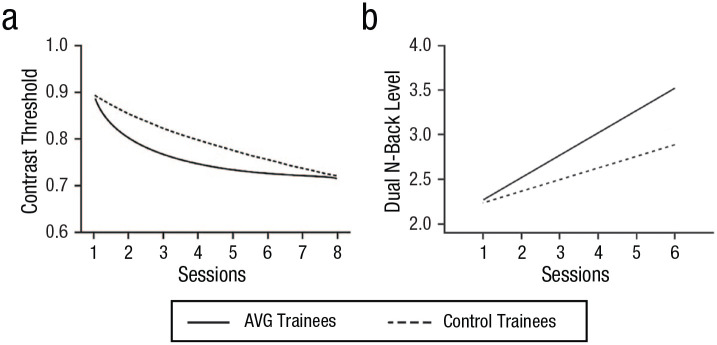
Impact of video game training on learning for participants assigned to either action or control video game play training. Learning was measured at posttest after each group had completed their respective training (45 hr long over 8 to 12 weeks). As shown by the two plots, the performance (i.e., learning) of the AVG-trained group improved more quickly than the performance of the control-trained group: AVG trainees could (a) perform the perceptual learning task with less and less contrast, showing faster learning than control trainees (lower means better performance), and (b) monitor for greater and greater values of *n* in the *n*-back task showing again faster learning than control trainees (higher *n*-back levels means better performance). All solid and dashed lines indicate the best fitting power functions replotted from Zhang et al. (2021). AVG = action video game.

Specifically, in an initial study and then a preregistered follow-up, participants trained on either AVGs or control video games for a total of 45 hr (about 1 hr per day for 5 days per week over a period of approximately 3 months). Participants in the two groups began video game training with comparable perceptual and executive function skills at pretest. After their video game training, however, posttest assessments revealed faster learning in AVG trainees compared with control video game trainees in both a perceptual task and an executive function task (Fig. 4).

And although the above study is the one long-term intervention study, to our knowledge, to explicitly examine the extent to which AVG training induces the learning-to-learn form of generalization, several cross-sectional studies have reported results consistent with learning to learn rather than immediate transfer, with AVG trainees learning perceptual, motor, or visuomotor sequence tasks more quickly than non-AVG trainees (Bejjanki et al., 2014; Romano Bergstrom et al., 2012).

## From the Lab to the Classroom: Example of Learning to Read

One major question concerns the types of real-world situations that might benefit from the enhancements induced by AVG experience. One such domain is learning to read, in which a variety of publications have suggested that AVG play facilitates reading acquisition in both typically developing and dyslexic children. Given the type of changes induced by AVG experience, it is perhaps not surprising that the theory underlying these empirical results is rooted in work highlighting the importance of attentional and executive skills in reading acquisition (Gori & Facoetti, 2015). For instance, research has characterized two primary attentional components essential for learning to read: the deployment of visual attentional skills to the page and covert attentional shifting abilities. In deep orthographies such as English, in which the mapping between letters and sounds suffers from many irregularities, the deployment of visuospatial attentional skills may still be relevant as the reader needs to solve a rather complex letter-to-sound mapping task (Bosse et al., 2007). Additionally, attentional shifting abilities have been proposed to mediate pseudoword reading and the use of phonological decoding strategies (Vidyasagar & Pammer, 2010) because they enable the implementation of sequential orthographic parsing strategies that facilitate the conversion of print to sounds. Although the exact attentional mechanisms by which these various abilities may affect the acquisition of literacy remains an issue of debate in the field, there is good agreement on the importance of enhanced attentional control in mastering the novel demands that reading acquisition puts on the learner, again highlighting the role of attention control in learning to learn.

**Fig. 5.**
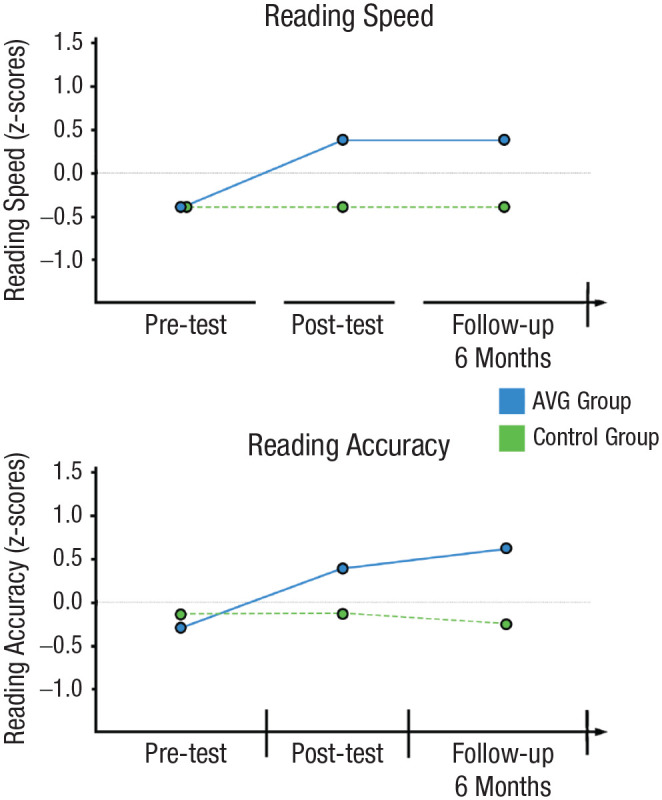
Impact of a purposefully designed action-like game on reading ability. Although both groups showed similar performance before training, the experimental group (in blue) showed greater improvement in reading speed (top) and accuracy (bottom) compared with the control group (in green) after training, which was still visible at a 6-month follow-up. Data replotted from Pasqualotto, Altarelli, et al. (2022).

In one specific recent test of this hypothesis (Pasqualotto, Altarelli, et al., 2022; Fig. 5), a child-friendly AVG was developed and contrasted against the educational game Scratch (control game) in 160 typically developing Italian-speaking children. Children played their respective game at school for two sessions of 1 hr per week over a period of 6 weeks for a total of 12 hr. A paper-and-pencil test of attention (barrage task—a sort of search-for-Waldo task) indicated increased attention after the AVG play compared with the control (Scratch) group, as expected. In addition, children trained on the AVG showed improved reading skills, as measured by word and pseudoword reading abilities after training, with the gains in attention explaining up to 15% of the variance in reading speed. The enhancement was, however, not limited to reading speed but extended to reading accuracy and was maintained at a 6-month follow-up date. These results mirror what has been found in developmental dyslexia, in which AVG play has been observed to enhance attentional control and reading skills in Italian-speaking dyslexic children (Bertoni et al., 2021; Franceschini et al., 2013).

Of final note is the finding that grades in Italian as measured by teachers in the classroom also improved in the AVG-trained group compared with the control-trained group. Critically, however, although better reading skills could be noted immediately after the completion of training when using more sensitive laboratory tasks, enhancements in grades were noted only 12 to 18 months after the end of training. Such delayed effects call for training studies that continue assessing the long-term impact of training at a scale of years rather than days, weeks, or months (Rebok et al., 2014).

## Caveats and Future Issues

The positive impact of AVG play is quite broad, generalizing to tasks that are quite dissimilar on the surface from video games. Indeed, there are few similarities between doing thousands of trials of a Gabor orientation discrimination task alone in a laboratory booth versus playing a first-person shooter game with friends at home. Yet the impact of AVGs nonetheless certainly rests on shared cognitive constructs between AVGs, which are quite rich and centrally tap core executive functions such as attentional control, and the generalization tasks considered. Furthermore, the impact of AVGs relies on the engaged neural subsystems being subject to reasonable plasticity. This might, for instance, explain why there is a paucity of studies documenting benefits to higher cognitive skills such as long-term planning or reasoning (which are often less important in more reaction-based AVGs). Similarly, meta-analyses suggest lesser (to no) impact on inhibitory control functions or to bottom-up attention, despite these functions certainly being called on during game play, which might be expected if those tasks are more strongly mediated by subcortical neural structures that are less easy to modify via experience, especially past their sensitive period of development.

Furthermore, as many mechanisms underlying human behavioral performance inherently trade off with each other, knowing in which tasks AVG experience may result in worse performance would be of great practical and theoretical significance. In many training domains, transfer is thought to be substantially mediated by a shared statistical structure (Klahr & Chen, 2011), and so-called negative transfer is a hallmark of structure learning that occurs when the statistics of new tasks violate those learned in previous tasks (Kattner et al., 2017). The failure thus far to find tasks in which AVGs underperform provides additional evidence in favor of a more attention-control-based mechanism because this mechanism is unlikely to make performance truly worse on novel tasks.

Finally, an important direction concerns specifying the key “ingredients” of AVGs. Here we have proposed that the unique combination of a demand to respond under time pressure, a high load on divided attention, and the requirement to flexibly shift attentional states on demand between divided and focused attention is central to the action mechanics (Pasqualotto, Parong, et al., 2022). It is already the case that the expanding space of commercial video games has directed us to define an action-like video game space that encompasses driving, real-time strategy, and multiplayer online battle arena as game genres likely to show similar impacts as AVGs (Bowman et al., 2024).

## Concluding Remarks

Together this work contributes to our theoretical understanding of the mechanisms that underlie cognitive enhancements while also offering practical suggestions for future applied research, including the most promising processing stages to target for broad cognitive enhancements, the duration that an intervention should last given its targeted domain of cognition, and/or the neural networks that may be the target of transcranial stimulation in combination with interventions based on video games to augment efficacy.

## Recommended Reading

Bediou, B., Rodgers, M. A., Tipton, E., Mayer, R. E., Green, C. S., & Bavelier, D. (2023). (See References). Examines the impact of action video games on cognition and discusses many of the main issues in the field (e.g., with respect to the link between training duration and effect size).

Franceschini, S., Gori, S., Ruffino, M., Viola, S., Molteni, M., & Facoetti, A. (2013). (See References). Excellent example of the potential (and perhaps expected by many) real-world positive impact of action video games on reading abilities.

Zhang, R. Y., Chopin, A., Shibata, K., Lu, Z.-L., Jaeggi, S. M., Buschkuehl, M., Buschkuehl, M., Green, C. S., & Bavelier, D. (2021). (See References). Demonstrates that action video-game play induces the “learning-to-learn” form of generalization; also exemplifies the methodological changes that need to be made to evaluate whether learning to learn is present.

## Transparency

*Action Editor:* Robert L. Goldstone

*Editor:* Robert L. Goldstone

*Declaration of Conflicting Interests*

The author(s) declared that there were no conflicts of interest with respect to the authorship or the publication of this article.
